# Role of Procurement Practice on Early Allograft Dysfunction in Liver Transplantation: A Propensity-Weighted Single-Center Analysis

**DOI:** 10.3390/jcm14207409

**Published:** 2025-10-20

**Authors:** Quirino Lai, Licia Iannello, Alice Viscione, Fabio Melandro, Giulia Diamantini, Silvia Quaresima, Flaminia Ferri, Stefano Ginanni Corradini, Gianluca Mennini, Massimo Rossi

**Affiliations:** 1 General Surgery and Organ Transplantation Unit, Department of General and Specialty Surgery, Sapienza University of Rome, Azienda Ospedaliero-Universitaria Policlinico Umberto I, 00161 Rome, Italy; licia.iannello@uniroma1.it (L.I.); viscione.alice@gmail.com (A.V.); fabio.melandro@uniroma1.it (F.M.); gldiamantini@gmail.com (G.D.); silvia.quaresima@uniroma1.it (S.Q.); gianluca.mennini@uniroma1.it (G.M.); massimo.rossi@uniroma1.it (M.R.); 2 Department of Translational and Precision Medicine, Sapienza University of Rome, Azienda Ospedaliero-Universitaria Policlinico Umberto I, 00161 Rome, Italy; flaminia.ferri@uniroma1.it (F.F.); stefano.corradini@uniroma1.it (S.G.C.)

**Keywords:** brain death donor, combined perfusion, expanded criteria donor, liver procurement, organ preservation

## Abstract

**Background/Objectives**: Liver transplantation (LT) remains the standard treatment for end-stage liver disease. While donation after brain death (DBD) is the predominant source of grafts, non-standard donors are increasingly used. Optimizing procurement techniques may improve graft function and reduce early allograft dysfunction (EAD). **Methods**: This retrospective monocenter study analyzed 231 first LT performed between 2013 and 2024. Patients were divided into two eras: Era 1 (n = 143, 2013–2019, standard aortic perfusion) and Era 2 (n = 88, 2019–2024, refined procurement strategies including combined aortic-portal perfusion, adjusted perfusion volumes, and additional caval venting). Exclusion criteria were retransplantation, DCD, split grafts, combined transplants, and early thrombosis. The primary endpoint was EAD. Secondary endpoints included graft loss and mortality. Stabilized inverse probability of treatment weighting (IPTW) was applied to balance groups. **Results**: After IPTW, EAD incidence was significantly reduced in Era 2 (42.3% vs. 24.6%, *p* < 0.0001). Similarly, graft loss (12.6% vs. 32.2%, *p* < 0.0001) and mortality (11.6% vs. 30.8%, *p* < 0.0001) decreased. Kaplan–Meier analysis showed improved graft survival in Era 2 (HR = 0.52, 95%CI: 0.28–0.99, *p* = 0.046). Sub-analysis of expanded criteria donors confirmed significant reductions in EAD, graft loss, and mortality. **Conclusions**: Refined procurement strategies in DBD grafts significantly reduced EAD, graft loss, and mortality. These simple, cost-effective refinements represent a valuable approach to optimize outcomes, particularly with marginal donors, and warrant validation in multicenter prospective studies.

## 1. Introduction

Liver transplantation (LT) is the definitive therapy for many causes of end-stage liver disease [1]. Consequently, the need to increase the number of available organs for transplantation is a key objective for physicians working in this field [1].

In recent years, several approaches have been adopted to expand graft availability, including donors after cardiac death (DCD) [2], the wider adoption of perfusion machines [3], and the growing interest in living donation [4]. Nevertheless, donation after brain death (DBD) still accounts for the majority of grafts in Western countries. Consequently, the refinement of surgical strategies aimed at achieving rapid and robust graft function recovery is fundamental, especially when non-standard DBD grafts are used to expand the donation pool [5].

Among the strategies we considered the following: (a) the simultaneous aortic–portal perfusion [6], (b) the use of a standardized amount of perfusion solution for flushing the organ [7], and (c) infrarenal caval venting when thoracic organs are procured [8].

The study aimed to explore whether the introduction of these specific procurement-related approaches reduced early allograft dysfunction (EAD). The effect was evaluated by comparing two 6-year eras. To minimize potential selection biases between the two eras, we used stabilized inverse probability of treatment weighting (IPTW).

## 2. Materials and Methods

### 2.1. Study Design

This retrospective, single-center observational study examined the outcomes of patients receiving a first LT. Approval was obtained from the Local Ethics Board of Sapienza University of Rome, and the study followed the Strengthening the Reporting of Observational Studies in Epidemiology (STROBE) guidelines.

### 2.2. Setting and Population

The LT center at Sapienza University of Rome, AOU Policlinico Umberto I, Rome, Italy, participated in this study. A total of 284 LTs were performed during the period 1 January 2013–31 December 2024. Exclusion criteria for the final analysis were: (a) retransplantation (n = 17), (b) graft from a DBD perfused with machine perfusion before implantation (n = 11), (c) split liver (n = 9), (d) graft from a controlled DCD (n = 8), (e) combined transplantation (n = 4), and f) early (≤7-day) hepatic artery/portal vein thrombosis (n = 4). After exclusions, 231 cases were enrolled in the final analysis. A total of 143 patients (61.9%) were in Era 1 (No refined procurement strategies: 1 January 2013–30 June 2019), while 88 patients (38.1%) were in Era 2 (Refined procurement strategies: 1 July 2019–31 December 2024).

### 2.3. Outcomes

The primary outcome was the rate of EAD. Secondary outcomes included (a) primary non-function (PNF)/delayed non-function (DNF), (b) graft loss, (c) patient death, and (d) retransplantation. A sub-analysis was also performed with the same outcomes in the cohort of patients transplanted with grafts derived from expanded criteria donors (ECD) [9]. The last follow-up date was 31 May 2025.

### 2.4. Data Collection

Data were extracted retrospectively from patient records. Data integrity was overseen by the study group’s Data Manager (QL), who resolved data errors and missing values through queries when feasible.

### 2.5. Definitions

ECD was defined according to the Eurotransplant definition: (a) donor age > 65 years; (b) ICU stay with ventilation > 7 days; (c) BMI > 30; (d) steatosis of the liver > 40%; (e) serum sodium > 165 mmol/L; (f) ALT > 105 U/L, AST > 90 U/L; (g) serum bilirubin > 3 mg/dL [9]. EAD was defined according to the Olthoff criteria [10]. PNF was defined as the immediate and irreversible failure of the transplanted liver graft, in the absence of identifiable technical or immunological causes of graft failure, necessitating urgent retransplantation or leading to patient death within the first 7 days post-transplantation. DNF was defined as the irreversible failure of the transplanted liver graft, in the absence of identifiable technical or immunological causes of graft failure, necessitating urgent retransplantation or leading to patient death after the first 7 days post-transplantation.

The Donor Risk Index (DRI) was calculated according to the original formulation by Feng et al. [11], integrating multiple donor- and graft-related variables into a composite risk score. A DRI value above 2.0 was considered indicative of a high-risk graft.

Urgent LT was defined as a transplant performed for acute liver failure (national super-urgency) or for acute-on-chronic liver failure (macroarea urgency).

### 2.6. Organ Procurement and Liver Transplantation Surgical Approaches

During Era 1, liver procurement was performed using aortic perfusion alone. A standard 5 L flush with Celsior^®^ (Institut Georges Lopez, Lissieu, France) was administered, with standard venous venting at the suprahepatic inferior vena cava. Following an internal quality audit and a multidisciplinary review of current literature, our center introduced in July 2019 (i.e., the beginning of Era 2) a unified “procurement optimization bundle” comprising three standardized measures: (a) simultaneous aortic and portal perfusion, (b) weight-adjusted perfusion volumes, and (c) systematic infrarenal caval venting during thoracic retrieval. The decision to implement these modifications simultaneously was pragmatic, aiming to harmonize retrieval practices across surgical teams and minimize inter-operator variability rather than to test individual components. Adherence was 100%, which was achievable thanks to the straightforward implementation and integration of the bundle within the routine donor management workflow. Portal perfusion consisted of a standard 2 L flush, while aortic perfusion was administered at a dose of 1 L of solution per 10 kg of donor body weight. During this period, both Celsior^®^ (Institut Georges Lopez, Lissieu, France) and IGL-1^®^ (Institut Georges Lopez, Lissieu, France) solutions were employed. As for the other aspects of the surgical techniques, in both eras, the hilar dissection was minimal, with the exclusive section of the common bile duct; the liver was procured en bloc with pancreas, spleen, and duodenum.

Liver transplantation in both eras was performed by the same surgical team, following a standardized technique. Caval reconstruction was carried out using a side-to-side piggyback approach. Biliary continuity was restored by duct-to-duct anastomosis, systematically protected with a T-tube.

### 2.7. Statistical Analysis

Continuous variables were presented as medians and first–third quartiles (Q1–Q3), while categorical variables were presented as counts and percentages. Categorical comparisons used Fisher’s exact test or chi-square test as appropriate, while the Mann–Whitney U test was used for continuous data. Missing data accounted for less than 10% of cases and were imputed using the median-of-nearby-points approach due to the skewed distribution of variables.

The cohort was divided into two eras (Era 1 vs. Era 2). Given the study’s non-randomized retrospective design, stabilized inverse probability of treatment weighting (IPTW) was applied to balance the groups [12].

Propensity scores were calculated for each patient based on multivariable logistic regression. Twenty-six relevant confounders were included as covariates: recipient weight, recipient bilirubin, recipient INR, hepatocellular carcinoma (HCC) as cause of LT, HCV as cause of LT, HBV as cause of LT, alcohol as cause of LT, acute liver failure as cause of LT, other pathology as cause of LT, cold ischemia time, warm ischemia time, extracorporeal circulation, donor weight, trauma as cause of donor death, anoxia as cause of donor death, cardiovascular accident as cause of donor death, donor dyslipidemia, other donor comorbidities, donor alcohol use, donor cardiac arrest(s), use of dopamine in the donor, use of adrenaline in the donor, donor ALT, donor GGT, donor sodium, liver graft fibrosis.

Stabilized weights were derived as follows:

SW = p/PS for the treated (Era 2) group, and SW = (1 − p)/(1 − PS) for the control (Era 1) group, where p is the marginal probability of treatment assignment and PS is the propensity score.

Standardized mean differences (SMDs) were calculated to report effect sizes, with thresholds of |SMD| < 0.1 indicating acceptable balance. A Love plot illustrated covariate balance before and after IPTW.

The post-IPTW pseudo-population was evaluated for different post-LT variables: AST peak within 7 days, ALT peak within 7 days, total bilirubin value at day 7, INR value at day 7, intensive care unit (ICU) stay, total length of stay (LOS), comprehensive complication index (CCI), PNF/DNF, EAD, death, graft loss, and retransplantation.

Kaplan–Meier survival analyses and the log-rank test were used for survival comparisons, with statistical significance set at *p* < 0.05. SPSS version 27.0 (IBM Corp., Armonk, NY, USA) and R 4.5.0 (R Foundation for Statistical Computing, Vienna, Austria) were used for analysis.rficial text editing (e.g., grammar, spelling, punctuation, and formatting) does not need to be declared.

## 3. Results

Donor- and recipient-related characteristics are shown in Table 1 and Table 2. The cohort comprised 231 cases, with a median follow-up of 60 months (Q1–Q3 = 17–107). The entire cohort was divided into two groups, namely Era 1 (before procurement implementation strategies: n = 143, 61.9%) and Era 2 (after procurement implementation strategies: n = 88, 38.1%).

Among the donor characteristics, most variables, including age, comorbidities, ECD incidence, hemodynamic instability, liver function tests, and steatosis, were not significantly different between the two groups, suggesting overall similarity in donor profiles (Table 1). Only a few statistically significant differences were observed. For example, more donors died due to anoxia (10.2% vs. 1.4%, *p* = 0.003), and a lower proportion of graft biopsies was observed in Era 2 (37.5% vs. 54.5%, *p* = 0.017). Among the histological features, the presence of fibrosis was more common in Era 2 (27.3% vs. 10.3%, *p* = 0.047) (Table 1).

When comparing the two eras in terms of recipient-related characteristics, an overall similarity was observed in their profiles (Table 2).

Postoperatively, Era 2 patients exhibited significantly lower bilirubin and INR values at day 7 (bilirubin: median 5.2 vs. 7.4 mg/dL, *p* < 0.0001; INR: median 1.16 vs. 1.25, *p* < 0.0001), while the peak AST within the first 7 days was higher (median: 853 vs. 540 IU/L, *p* = 0.002). Additionally, the CCI was significantly higher in Era 2 (median: 29.6 vs. 20.9, *p* = 0.023). Outcome indicators such as EAD, patient death, and graft loss were significantly lower in Era 2. The incidence of EAD declined from 43.4% to 28.4% (*p* = 0.033), mortality from 32.2% to 15.9% (*p* = 0.010), and graft loss from 34.3% to 17.0% (*p* = 0.007) (Table 2).

### 3.1. Stabilized Inverse Probability of Treatment Weighting Results

To mitigate bias from the retrospective, non-balanced design, a stabilized IPTW approach was performed. In the comparison of donors, recipients, and procedural variables across the two eras, the SMDs revealed several notable findings (Table 3). Before weighting, substantial imbalances were observed in some key variables, such as the use of inotropes in donors, anoxia as a cause of death, and warm ischemia time. After weighting, the covariates showing only small effect sizes (|SMD| < 0.2) increased from 15/26 (57.7%) to 21/26 (80.8%), suggesting adequate balance between the two groups for the majority of variables (Table 3).

**Figure 1:**
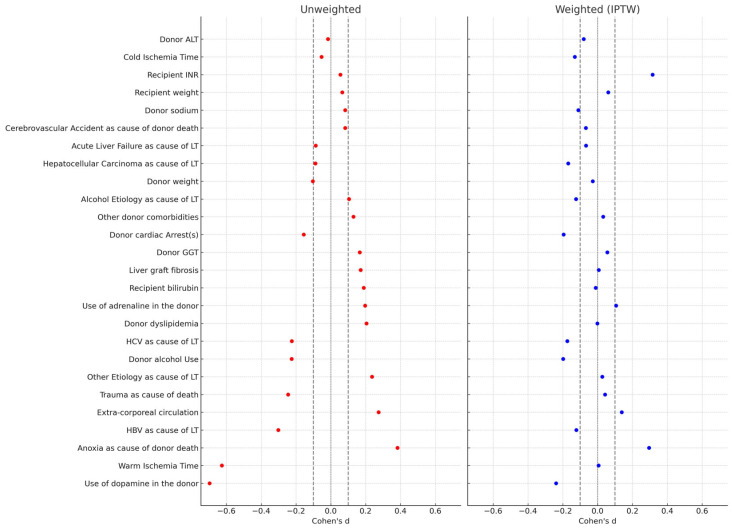
Love Plot comparing the covariate balancing before and after the stabilized IPTW.

The results were also visualized in the Love plot (Figure 1).

### 3.2. Clinical Outcomes After Stabilized Inverse Probability of Treatment Weighting

After stabilizing IPTW, a pseudo-population of 208 transplanted cases in Era 1 vs. 207 in Era 2 was generated. Several significant differences were observed in post-transplant biochemical parameters and clinical outcomes between the two eras after weighting.

Median peak AST levels were significantly higher in Era 2 (624 vs. 540 U/L; *p* = 0.002). On the other hand, total bilirubin at day 7 (4.7 vs. 7.1 mg/dL; *p* = 0.0001) and INR (1.17 vs. 1.26; *p* < 0.0001) were markedly lower in Era 2. CCI was significantly higher in Era 2 (29.6 vs. 20.9; *p* = 0.02).

In terms of clinical outcomes, the incidence of EAD was significantly reduced in Era 2, decreasing from 42.3% to 24.6% (*p* < 0.0001). Similar results were observed for PNF/DNF incidence (2.4% vs. 7.7%; *p* = 0.03). Graft loss (12.6% vs. 32.2%; *p* < 0.0001) and overall mortality (11.6% vs. 30.8%; *p* < 0.0001) were significantly lower in Era 2. There was no statistically significant difference in retransplantation rates (2.9% vs. 5.8%; *p* = 0.23) (Table 4).

**Figure 2:**
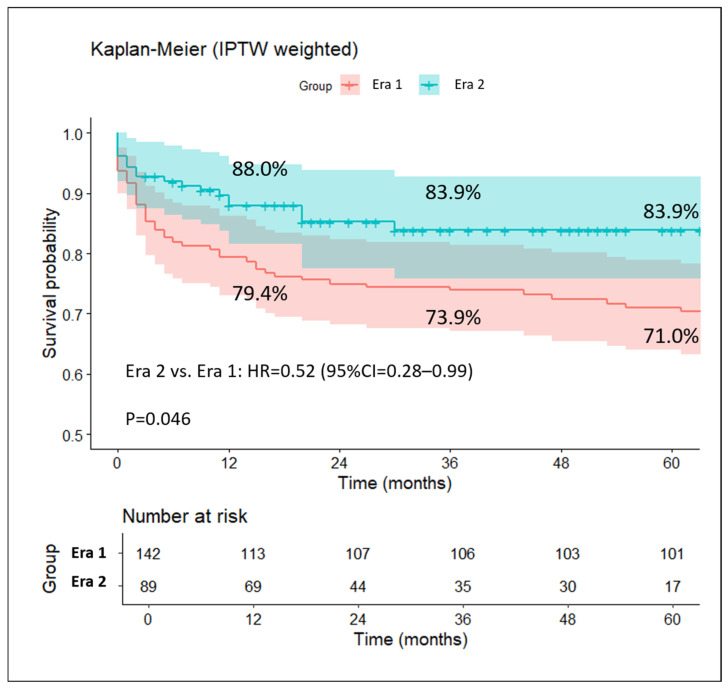
Survival curves in the two eras after the stabilized IPTW.

A post-IPTW weighted Kaplan–Meier survival analysis was performed to compare graft survival rates between the two eras. Patients in Era 2 showed significantly improved graft survival compared to those in Era 1, with a univariable hazard ratio (HR) of 0.52 (95% CI: 0.28–0.99; *p* = 0.046). These findings indicate a 48% reduction in the hazard of graft loss in Era 2 (Figure 2).

### 3.3. Sub-Analysis Focused on ECD

In the ECD-only sub-analysis, several significant differences were observed. The incidence of EAD was significantly reduced in Era 2, decreasing from 43.5% to 27.9% (*p* = 0.02). Graft loss (18.8% vs. 35.9%; *p* = 0.005) and overall mortality (16.5% vs. 35.9%; *p* = 0.002) were significantly lower in Era 2 (Table 4).

### 3.4. Sub-Analysis Focused on Non-Urgent Recipients

In the non-urgent LT sub-analysis, several significant differences were also reported. The incidence of EAD was even inferior in Era 2, decreasing from 40.3% to 21.1% (*p* < 0.001). PNF/DNF (1.3% vs. 6.0%; *p* = 0.001), graft loss (12.7% vs. 26.7%; *p* < 0.001), and overall mortality (11.4% vs. 25.6%; *p* < 0.001) were significantly lower in Era 2 (Table 4).

## 4. Discussion

In the present study, we investigated the impact of refined surgical procurement strategies in DBD grafts on early and late outcomes after liver transplantation. By comparing two consecutive eras in a single-center experience, we showed that the adoption of combined aortic–portal perfusion, adjustment of perfusion volumes, and systematic double caval venting during concomitant procurement of thoracic organs were associated with a significant reduction in the incidence of EAD, graft loss, and patient mortality. Importantly, these findings remained consistent after applying stabilized IPTW, thereby reinforcing the robustness of the results and minimizing potential confounding related to the non-randomized design. Our results align with prior reports suggesting that procurement techniques play a crucial role in graft quality and post-transplant outcomes.

While most of the attention in recent years has been devoted to DCD, machine perfusion technologies, or living donation [2,3,4], the optimization of DBD procurement protocols remains an essential step to expand the donor pool and maximize graft utilization.

As for dual vs. aortic-only perfusion, previous studies reported mixed results for post-LT outcomes [6,13,14,15]. A meta-analysis reported no substantial differences between the two procedures for PNF rate, biliary complications, or 1-year graft survival [13]. However, a study from the Australia/New Zealand Liver Transplant Registry, including 1382 LTs (957 aortic-only; 425 dual perfusion), reported a trend toward improved outcomes when dual perfusion was used, which became significant when considering higher-risk donors alone (graft survival: hazard ratio (HR) = 0.49; 95% CI = 0.26–0.92) [14].

An Italian study investigated double perfusion as a tool for minimizing biliary damage in very old DBD donors (≥80 years): aortic-only perfusion was associated with 13.4% vs. 1.9% ischemia-type biliary lesions (P = 0.008), while double perfusion was a relevant protective factor (HR = 0.1; P = 0.04) [6].

A randomized prospective study from Italy also compared the two perfusion techniques in suboptimal grafts (i.e., age ≥ 60 years, steatosis > 20%, hemodynamic instability, peak transaminases): aortic-only perfusion cases reported significantly higher transaminase and bilirubin levels after LT, more PNF cases (35% vs. 5%; P = 0.01), and lower 6-month graft survival rates (58% vs. 100%) [15].

The results suggest that dual perfusion is particularly useful for older donor grafts. Although progressively increasing, this type of resource is still internationally limited. Some countries like Italy commonly use grafts from elderly DBDs [16,17], even procuring organs from centenarian donors [18]. The physiological rationale behind the findings is plausible. A combined aortic–portal flush ensures a more homogeneous distribution of the preservation solution across the parenchyma, avoiding areas of relative stasis. As a consequence, minimization of ischemia–reperfusion injury is expected, allowing homogeneous and rapid preservation of the older donor graft [19].

Similarly, the use of adequate perfusion volumes and the implementation of multiple caval venting techniques have been sporadically reported as measures capable of facilitating better graft clearance. However, few studies have directly compared different procurement strategies in a systematic manner [20,21], and our analysis contributes novel evidence supporting their clinical value. In this case as well, the rationale is plausible: a higher volume of perfusion fluid contributes to more effective removal of cellular debris and blood elements, thereby reducing microthrombosis and early ischemia-related injury. Finally, the addition of infrarenal caval venting, particularly in the context of multiorgan procurement, reduces intravascular resistance and facilitates a rapid and uniform washout.

The higher early transaminase peaks observed in Era 2 most likely reflect reperfusion-related enzyme release rather than impaired hepatocellular function. This interpretation is supported by the concomitant lower bilirubin and INR values at day 7. Such evidence was also reported in the meta-analysis by Hameed et al., in which a higher peak of transaminases was recorded in dual- compared with aortic-only–perfused livers (standardized mean difference, 0.24; 95% confidence interval, 0.01–0.47) [13]. Alternatively, this trend could partly result from the greater use of marginal grafts in Era 2 (including donors after anoxia and grafts with increased fibrosis), together with a lower biopsy rate, reflecting a broader acceptance policy. Fibrosis was indeed more frequently observed among biopsied grafts in Era 2, likely reflecting a progressive shift toward accepting older and extended-criteria donors, together with a lower overall biopsy rate that may have introduced sampling variability. Nevertheless, fibrosis was included in the IPTW model and remained well balanced after weighting, suggesting that this factor did not influence the observed differences in outcomes between eras.

The most striking finding of this study is the significant reduction in EAD from 42.3% in Era 1 to 24.6% in Era 2 after IPTW adjustment (*p* < 0.0001). In the original study from Olthoff, the EAD incidence was 23.2% [10]. However, in several cohorts applying the Olthoff definition, EAD rates varied widely by donor type and graft quality [22,23,24]. In a cohort of 1068 LT patients from Toronto, the incidence of EAD was 44%, with 71% vs. 41% in DCD versus DBD cases [22]. A study from the Mayo Clinic Arizona explored 611 LT cases using non-conventional grafts, reporting 52.2% of EAD cases (79% in DCD grafts and 40% in DBD grafts) [23]. A study from Germany performed on 621 cases reported 53.6% of EAD [24]. In Italy, the use of marginal grafts is frequent [25]. Also in the present series, approximately 60% of cases were transplanted using ECD grafts, justifying the high EAD value in Era 1. Thus, achieving an 18 percentage-point absolute reduction was clinically meaningful, bringing our results back in line with international single-center benchmarks [10]. In the specific sub-cohort of ECDs, a similar 15 percentage-point absolute reduction was reported, confirming the validity of our strategies in minimizing EAD also in the subgroup of marginal grafts. Even better, considering only the non-urgent LTs, the EAD percentage reduced from 40.3 to 21.1%.

The correlation between EAD and long-term outcomes has been well reported in several studies [10,22,23,24]. Also in this series, the positive impact in terms of graft and patient survival was observed, with a relevant decrease in mortality and graft loss. Although such a result may be partially influenced by the shorter follow-up of Era 2, a 10% increase in graft survival reported at 1 and 3 years after LT represents a relevant improvement, further suggesting the importance of the implemented strategies adopted for graft management during procurement.

Despite the relative homogeneity of the two cohorts investigated and the rigorous use of statistical methods (i.e., IPTW) aimed at further minimizing potential differences, some limitations of the study must be acknowledged. First, its retrospective and single-center design inherently carries the risk of bias, although the use of IPTW considerably mitigates this issue. Second, improvements in perioperative care and recipient management over time may have contributed to the observed survival benefit, even if donor and recipient characteristics were overall well balanced between eras. Third, the sample size, although relatively large for a single center, remains limited compared with multicenter registry analyses. Fourth, the three technical refinements were introduced as a single, center-wide protocol, which precluded isolation of the specific contribution of each individual component to the observed outcomes. However, the bundle was designed as a harmonization strategy based on converging mechanistic and empirical evidence supporting the safety and physiological rationale of each element. Finally, we did not directly compare these strategies with the emerging perfusion machine technologies, which currently represent a major innovation in the field. We are confident that the use of perfusion machines should further improve management quality, decreasing EAD incidence [26]. Moreover, donor-to-recipient matching may also play a relevant role in post-transplant outcomes, particularly in settings where marginal or extended-criteria donors are frequently used. This aspect, not specifically addressed in the present analysis, has been extensively discussed in the recent literature [27,28].

## 5. Conclusions

Our study highlights that relatively simple refinements in procurement technique can lead to meaningful improvements in graft function and survival after LT. These findings underscore the importance of optimizing surgical protocols in DBD donation, particularly when using non-standard grafts, as an accessible and cost-effective approach to expand the donor pool. Future multicenter and prospective studies are warranted to validate these results and to position procurement refinements within the broader context of evolving technologies such as normothermic regional perfusion and ex situ machine perfusion.

## Figures and Tables

**Table 1:** Donor-related characteristics.

Variable	Era 1 (143, 61.9%)	Era 2 (88, 38.1%)	*p*-Value
	N (%) or Median (Q1–Q3)		
Male sex	69 (48.6)	43 (48.9)	1.00
Age, years	56.5 (43.9–68.4)	58.0 (46.0–66.3)	0.82
Weight, kg	70.0 (65.0–80.0)	70.0 (65.0–77.3)	0.39
Height, cm	168.0 (160.0–175.0)	167.0 (160.8–175.0)	0.39
BMI	25.4 (23.4–27.8)	25.0 (24.0–27.0)	0.95
ICU stay, days	3 (2–6)	3 (2–6)	0.29
National organ sharing	68 (47.6)	42 (47.7)	1.00
African ethnicity	1 (0.7)	1 (1.1)	1.00
Cause of death			
Trauma	35 (24.5)	13 (14.8)	0.11
Anoxia	2 (1.4)	9 (10.2)	0.003
CVA	102 (71.3)	66 (75.0)	0.65
Other	1 (0.7)	2 (2.3)	0.56
T2DM	13 (9.1)	9 (10.2)	0.96
Arterial hypertension	59 (41.3)	33 (37.5)	0.67
Cardiopathy	29 (20.3)	23 (26.1)	0.38
Dyslipidemia	17 (11.9)	17 (19.3)	0.18
Smoking	54 (37.8)	33 (37.5)	1.00
Alcohol abuse	10 (7.0)	2 (2.3)	0.14
HBV Anticore positive	11 (7.7)	7 (8.0)	1.00
Severe hypotension(s)	19 (13.4)	17 (19.3)	0.30
Cardiac arrest(s)	24 (16.9)	10 (11.4)	0.34
Noradrenaline	113 (79.0)	74 (84.1)	0.44
Adrenaline	6 (4.2)	8 (9.1)	0.22
Dopamine	32 (22.4)	1 (1.1)	<0.0001
Dobutamine	7 (4.9)	4 (4.5)	1.00
AST last value, IU/L	31 (23–52)	30 (17–39)	0.074
AST peak, IU/L	34 (25–59)	34 (26–57)	0.66
ALT last value, IU/L	26 (17–46)	23 (13–36)	0.08
ALT peak, IU/L	29 (18–49)	28 (16–49)	0.39
INR last value	1.15 (1.02–1.29)	1.15 (1.08–1.27)	0.63
Total bilirubin last value, mg/dL	0.6 (0.4–0.9)	0.6 (0.4–1.0)	0.21
Total bilirubin peak, mg/dL	0.7 (0.5–1.0)	0.7 (0.6–1.1)	0.087
GGT last value, IU/L	21 (14–44)	21 (14–56)	0.36
Biopsy of the graft	78 (54.5)	33 (37.5)	0.017
Macrosteatosis	5 (0–10)	1 (0–5)	0.19
Microsteatosis	14–445 (2–12)	3 (0–10)	0.12
Fibrosis	8 (10.3)	9 (27.3)	0.047
Necrosis	7 (9.0)	3 (9.1)	1.00
DRI	2.21 (1.46–3.71)	2.24 (1.58–3.60)	0.95
DRI > 2.0	78 (54.5)	49 (55.7)	0.97
ECD	87 (60.8)	53 (60.2)	1.00

**Abbreviations:** N, number; Q1–Q3, Quartile 1–Quartile 3; BMI, body mass index; ICU, intensive care unit; CVA, cerebrovascular accident; T2DM, type 2 diabetes mellitus; HBV, hepatitis B virus; AST, aspartate transaminase; ALT, alanine transaminase; INR, international normalized ratio; GGT, gamma-glutamyl-transferase; DRI, Donor Risk Index; ECD, expanded criteria donor.

**Table 2:** Recipient-related characteristics and post-LT outcomes (pre-stabilized IPTW results).

Variable	Era 1 (143, 61.9%)	Era 2 (88, 38.1%)	*p*-Value
	N (%) or Median (Q1–Q3)		
Male sex	124 (86.7%)	69 (78.4%)	0.14
Age, years	56.8 (47.7–61.7)	58.8 (50.7–64.1)	0.08
Weight, kg	76.0 (65.5–87.0)	79.8 (70.0–85.0)	0.74
Height, cm	170.0 (166.5–177.0)	174.5 (167.8–177.0)	0.34
BMI	26.0 (22.9–29.1)	26.1 (23.7–29.0)	0.88
Creatinine, mg/dL	0.9 (0.7–1.2)	0.8 (0.8–1.5)	0.07
Total bilirubin, mg/dL	3.1 (1.3–8.6)	1.0 (0.9–5.4)	0.001
INR	1.50 (1.28–2.02)	1.58 (1.15–1.70)	0.39
Sodium, mEq/L	138.0 (135.0–140.0)	137.5 (136.0–140.0)	0.82
MELD	16 (11–23)	14 (13–20)	0.87
HCC	73 (51.0)	41 (46.6)	0.60
HCV	45 (31.5)	19 (21.6)	0.14
HBV	28 (19.6)	8 (9.1)	0.051
HDV coinfection	6 (4.2)	2 (2.3)	0.71
Alcohol	56 (39.2)	39 (44.3)	0.53
MASLD	19 (13.3)	19 (21.6)	0.14
Acute liver failure	15 (10.5)	7 (8.0)	0.68
Other disease	13 (9.1)	15 (17.0)	0.11
Organ procurement in urgency	19 (13.3)	17 (19.3)	0.30
ECC	2 (1.4)	6 (6.8)	0.056
CIT, min	400 (385–435)	385 (385–422)	0.45
**Post-LT outcomes**			
AST peak within the first 7 days	540 (315–968)	853 (481–1278)	0.002
ALT peak within the first 7 days	472 (338–977)	457 (312–942)	0.69
Total bilirubin, mg/dL 7th day	7.4 (4.4–13.7)	5.2 (3.0–8.2)	<0.0001
INR, 7th day	1.25 (1.17–1.38)	1.16 (1.05–1.25)	<0.0001
ICU stay, days	7 (5–12)	8 (5–12)	0.089
Total LOS, days	18 (15–30)	22 (16–38)	0.055
CCI	20.9 (0.0–39.7)	29.6 (20.9–42.6)	0.023
PNF/DNF	11 (7.7)	2 (2.3)	0.14
EAD	62 (43.4)	25 (28.4)	0.033
Biliary complication	17 (11.9)	8 (9.1)	0.66
Ischemic cholangiopathy	11 (7.7)	6 (6.8)	1.00
Death	46 (32.2)	14 (15.9)	0.010
Graft loss	49 (34.3)	15 (17.0)	0.007
Re–transplantation	8 (5.6)	3 (3.4)	0.54

**Abbreviations:** N, number; Q1–Q3, Quartile 1–Quartile 3; BMI, body mass index; INR, international normalized ratio; MELD, model for end–stage liver disease; HCC, hepatocellular cancer; HCV, hepatitis C virus; HBV, hepatitis B virus; HDV, hepatitis D virus; MASLD, metabolic dysfunction–associated steatotic liver disease; ECC, extra–corporeal circulation; CIT, cold ischemia time; AST, aspartate transaminase; ALT, alanine transaminase; INR, international normalized ratio; ICU, intensive care unit; LOS, length of stay; CCI, comprehensive complication index; PNF, primary non–function; DNF, delayed non–function; EAD, early allograft dysfunction.

**Table 3:** Results of the stabilized IPTW.

Variables	Unweighted			Weighted		
	Era 1	Era 2	SMD	Era 1	Era 2	SMD
Recipient weight	77.08 ± 13.80	77.95 ± 12.61	0.07	77.43 ± 13.98	78.24 ± 12.58	0.06
Recipient bilirubin	0.70 ± 0.48	0.80 ± 0.59	0.19	0.71 ± 0.49	0.71 ± 0.50	−0.01
Recipient INR	1.20 ± 0.25	1.22 ± 0.33	0.05	1.21 ± 0.25	1.37 ± 0.68	0.32
HCC as a cause of LT	0.51 ± 0.50	0.47 ± 0.50	−0.09	0.51 ± 0.50	0.43 ± 0.49	−0.17
HCV as a cause of LT	0.31 ± 0.46	0.22 ± 0.41	−0.23	0.30 ± 0.46	0.22 ± 0.42	−0.17
HBV as a cause of LT	0.20 ± 0.40	0.09 ± 0.29	−0.30	0.19 ± 0.39	0.15 ± 0.35	−0.12
Alcohol as a cause of LT	0.39 ± 0.49	0.44 ± 0.50	0.10	0.40 ± 0.49	0.34 ± 0.47	−0.12
ALF as a cause of LT	0.10 ± 0.31	0.08 ± 0.27	−0.09	0.10 ± 0.30	0.08 ± 0.27	−0.07
Others as a cause of LT	0.09 ± 0.29	0.17 ± 0.38	0.24	0.10 ± 0.30	0.11 ± 0.31	0.03
Cold Ischemia Time	401.84 ± 68.44	398.52 ± 54.46	−0.05	398.03 ± 69.75	389.60 ± 58.32	−0.13
Warm Ischemia Time	64.15 ± 13.32	56.30 ± 11.59	−0.63	62.28 ± 12.64	62.36 ± 17.05	0.01
Extra-corporeal circulation	0.01 ± 0.12	0.07 ± 0.25	0.28	0.02 ± 0.13	0.04 ± 0.20	0.14
Donor weight	72.93 ± 12.49	71.74 ± 10.22	−0.10	72.53 ± 12.10	72.23 ± 9.69	−0.03
Trauma as a cause of donor death	0.24 ± 0.43	0.15 ± 0.35	−0.25	0.23 ± 0.42	0.25 ± 0.43	0.04
Anoxia as a cause of donor death	0.01 ± 0.12	0.10 ± 0.30	0.38	0.02 ± 0.12	0.08 ± 0.27	0.30
CVA as a cause of donor death	0.71 ± 0.45	0.75 ± 0.43	0.08	0.72 ± 0.45	0.69 ± 0.46	−0.07
Donor dyslipidemia	0.12 ± 0.32	0.19 ± 0.39	0.21	0.13 ± 0.33	0.13 ± 0.33	−0.00
Other donor comorbidities	0.01 ± 0.08	0.02 ± 0.15	0.13	0.01 ± 0.11	0.02 ± 0.13	0.03
Donor alcohol use	0.07 ± 0.26	0.02 ± 0.15	−0.23	0.06 ± 0.23	0.02 ± 0.14	−0.20
Donor cardiac arrest(s)	0.17 ± 0.37	0.11 ± 0.32	−0.16	0.15 ± 0.35	0.08 ± 0.28	−0.20
Use of dopamine in the donor	0.22 ± 0.42	0.01 ± 0.11	−0.70	0.17 ± 0.38	0.09 ± 0.29	−0.24
Use of adrenaline in the donor	0.04 ± 0.20	0.09 ± 0.29	0.20	0.04 ± 0.18	0.06 ± 0.23	0.11
Donor ALT	38.97 ± 59.12	38.08 ± 47.62	−0.02	37.01 ± 52.87	33.21 ± 40.88	−0.08
Donor GGT	43.15 ± 57.16	54.02 ± 72.55	0.17	43.74 ± 58.36	47.09 ± 61.57	0.06
Donor sodium	137.21 ± 5.48	137.60 ± 3.94	0.08	137.21 ± 5.54	136.67 ± 4.00	−0.11
Liver graft fibrosis	0.06 ± 0.23	0.10 ± 0.30	0.17	0.07 ± 0.25	0.07 ± 0.26	0.01

**Abbreviations:** SMD, standardized mean difference; INR, international normalized ratio; HCC, hepatocellular cancer; LT, liver transplantation; HCV, hepatitis C virus; HBV, hepatitis B virus; ALF, acute liver failure; CVA, cerebrovascular accident; ALT, alanine transaminase; GGT, gamma-glutamyl-transferase.

**Table 4:** Clinical outcomes after LT in the pseudo-population generated after stabilized IPTW and sub-analyses only focused on a) LTs performed using grafts coming from expanded criteria donors, and b) LTs performed in non-urgent recipients.

Variable	Era 1 (n = 208)	Era 2 (n = 207)	*p*-Value
AST peak	540.0 (285.0–935.0)	624.0 (428.0–1096.0)	0.002
ALT peak	444.0 (299.0–816.0)	382.0 (265.0–677.0)	0.69
Total bilirubin (7th day)	7.1 (4.4–13.6)	4.7 (3.2–7.5)	0.0001
INR (7th day)	1.26 (1.18–1.38)	1.17 (1.03–1.27)	<0.0001
ICU stay (days)	3 (2–6)	3 (2–5)	0.29
Total LOS (days)	18 (15–29)	22 (16–42)	0.055
CCI	20.9 (0.0–39.7)	29.6 (20.9–41.8)	0.02
PNF/DNF	16 (7.7%)	5 (2.4%)	0.03
EAD	88 (42.3%)	51 (24.6%)	<0.0001
Biliary complication	30 (14.4)	26 (12.6)	0.56
Ischemic cholangiopathy	27 (13.0)	18 (8.7)	0.16
Death	64 (30.8%)	24 (11.6%)	<0.0001
Graft loss	67 (32.2%)	26 (12.6%)	<0.0001
Re-transplantation	12 (5.8%)	6 (2.9%)	0.23
**Only cases with expanded criteria donors**			
**Variable**	**Era 1 (n = 124)**	**Era 2 (n = 99)**	***p*-value**
AST peak	540.0 (302.0–961.0)	874.0 (529.0–1320.0)	0.17
ALT peak	425.0 (299.0–840.0)	446.0 (318.0–970.0)	0.70
Total bilirubin (7th day)	8.2 (4.4–14.5)	5.6 (2.8–8.0)	0.005
INR (7th day)	1.3 (1.2–1.4)	1.2 (1.1–1.2)	<0.0001
ICU stay (days)	7 (5–15)	9 (6–12)	0.58
Total LOS (days)	18 (15–30)	22 (16–42)	0.19
CCI	20.9 (0.0–41.8)	29.6 (20.9–51.0)	0.53
PNF/DNF	8 (6.8%)	5 (4.7%)	0.51
EAD	54 (43.5%)	28 (27.9%)	0.02
Biliary complication	14 (11.1)	11 (11.4)	0.94
Ischemic cholangiopathy	9 (7.4)	8 (8.0)	0.88
Death	45 (35.9%)	16 (16.5%)	0.002
Graft loss	45 (35.9%)	19 (18.8%)	0.005
Re-transplantation	3 (2.2%)	6 (6.0%)	0.16
**Only non-urgent transplants**			
**Variable**	**Era 1 (n = 124)**	**Era 2 (n = 71)**	***p*-value**
AST peak	525.8 (282.5–929.3)	546.9 (407.8–1014.9)	0.006
ALT peak	421.2 (288.2–783.4)	367.6 (250.6–631.6)	0.060
Total bilirubin (7th day)	6.8 (4.2–12.6)	4.5 (3.1–7.5)	<0.001
INR (7th day)	1.25 (1.18–1.38)	1.18 (1.03–1.26)	<0.001
ICU stay (days)	6.00 (4.0–9.0)	8.00 (6.0–14.5)	<0.001
Total LOS (days)	6.00 (4.0–9.0)	8.00 (6.0–14.5)	<0.001
CCI	17.62 (15.0–28.0)	22.00 (16.0–40.9)	0.001
PNF/DNF	7/124 (6.0%)	1/71 (1.3%)	0.022
EAD	52/124 (40.3%)	19/71 (21.1%)	<0.001
Biliary complication	14/124 (11.8%)	8/71 (17.6%)	0.125
Ischemic cholangiopathy	10/124 (8.7%)	6/71 (15.6%)	0.048
Death	34/124 (25.6%)	12/71 (11.4%)	<0.001
Graft loss	36/124 (26.7%)	13/71 (12.7%)	<0.001
Re-transplantation	5/124 (4.0%)	2/71 (2.1%)	0.306

**Abbreviations:** AST, aspartate transaminase; ALT, alanine transaminase; INR, international normalized ratio; ICU, intensive care unit; LOS, length of stay; CCI, comprehensive complication index; PNF, primary non-function; DNF, delayed non-function; EAD, early allograft dysfunction.

## Data Availability

The data that support the findings of this study are not publicly available but can be obtained from the corresponding author upon reasonable request and subject to appropriate institutional approvals.

## Abbreviations

The following abbreviations are used in this manuscript:

ALT	Alanine aminotransferase
AST	Aspartate aminotransferase
BMI	Body mass index
CCI	Comprehensive complication index
CI	Confidence interval
DBD	Donation after brain death
DCD	Donation after cardiac death
DNF	Delayed non-function
EAD	Early allograft dysfunction
ECD	Expanded criteria donor
HCC	Hepatocellular carcinoma
HBV	Hepatitis B virus
HCV	Hepatitis C virus
HR	Hazard ratio
ICU	Intensive care unit
INR	International normalized ratio
IPTW	Inverse probability of treatment weighting
IQR	Interquartile range
LOS	Length of stay
LT	Liver transplantation
PNF	Primary non-function
PS	Propensity score
Q1–Q3	First–third quartile
SW	Stabilized weight
STROBE	Strengthening the Reporting of Observational Studies in Epidemiology
